# History of the York Dispensary, Containing an Account of Its Origin and Progress to the Present Time, Comprising a Period of Fifty-Seven Years

**Published:** 1845-07

**Authors:** 


Art. IV.—*History of the York Dispensary, containing an Account of its Origin and Progress to the present time, comprising a period of fifty-seven years.* By Oswald Allen.—*York,* 1845. 8vo, pp. 116.

History teaches the lessons of experience to those who will listen to it, and rightly interpret it. Although the history of the York Dispensary possesses chiefly a local interest, it cannot fail to be of value to those who intend to found a medical charity, as presenting a fair instance how a useful institution may arise from small beginnings, and showing clearly the nature of the difficulties that may be expected in maturing a medical charity, and how those difficulties may be surmounted. The author is the father of the profession in York, the treasurer of the charity, and the sole survivor of those who co-operated with him in founding the Dispensary. It is written in an excellent spirit, and proves that Mr. Allen enjoys a green old age, and that the retrospect of his useful life has not been made, at least, with painful feelings. These are neatly stated in a modest preface, and are such as might be expected from an intelligent practitioner who has practised his profession from a love thereof. We commend this little work to the notice of those who take a special interest in medical charities.

